# Social Media Platforms Listening Study on Atopic Dermatitis: Quantitative and Qualitative Findings

**DOI:** 10.2196/31140

**Published:** 2022-01-28

**Authors:** Paméla Voillot, Brigitte Riche, Michel Portafax, Pierre Foulquié, Anaïs Gedik, Sébastien Barbarot, Laurent Misery, Stéphane Héas, Adel Mebarki, Nathalie Texier, Stéphane Schück

**Affiliations:** 1 Kap Code Paris France; 2 Sanofi Genzyme Gentilly France; 3 Centre Hospitalier Universitaire de Nantes Nantes France; 4 Centre Hospitalier Universitaire de Brest Brest France; 5 Université de Rennes 2 Rennes France

**Keywords:** atopic dermatitis, Atopic Dermatitis Control Tool, health-related quality of life, social media use, real world, dermatology, skin disease, social media, online health information, online health, health care

## Abstract

**Background:**

Atopic dermatitis (AD) is a chronic, pruritic, inflammatory disease that occurs most frequently in children but also affects many adults. Social media have become key tools for finding and disseminating medical information.

**Objective:**

The aims of this study were to identify the main themes of discussion, the difficulties encountered by patients with respect to AD, the impact of the pathology on quality of life (QoL; physical, psychological, social, or financial), and to study the perception of patients regarding their treatment.

**Methods:**

A retrospective study was carried out by collecting social media posts in French language written by internet users mentioning their experience with AD, their QoL, and their treatments. Messages related to AD discomfort posted between July 1, 2010, and October 23, 2020, were extracted from French-speaking publicly available online forums. Automatic and manual extractions were implemented to create a general corpus and 2 subcorpuses depending on the level of control of the disease.

**Results:**

A total of 33,115 messages associated with AD were included in the analysis corpus after extraction and cleaning. These messages were posted by 15,857 separate web users, most of them being women younger than 40 years. Tips to manage AD and everyday hygiene/treatments were among the most discussed topics for controlled AD subcorpus, while baby-related topics and therapeutic failure were among the most discussed topics for insufficiently controlled AD subcorpus. QoL was discussed in both subcorpuses with a higher proportion in the controlled AD subcorpus. Treatments and their perception were also discussed by web users.

**Conclusions:**

More than just emotional or peer support, patients with AD turn to online forums to discuss their health. Our findings show the need for an intersection between social media and health care and the importance of developing new approaches such as the Atopic Dermatitis Control Tool, which is a patient-related disease severity assessment tool focused on patients with AD.

## Introduction

Atopic dermatitis (AD) is a chronic, pruritic, inflammatory skin disease that occurs most frequently in children but that can also affect adults. The course of the disease is relapsing, and it is frequently associated with elevated levels of serum immunoglobulin E, individual or family history of food allergies, allergic rhinitis, and asthma [1-3]. According to the World Health Organization Global Burden of Diseases initiative’s data, AD ranks 15th among all nonfatal disabilities worldwide and has the highest disease burden among skin diseases as measured by disability-adjusted life-years [4]. Childhood-onset AD begins early in life, with 50% diagnosed in the first year of life and 85% by 5 years of age [1,5]. However, AD can present at any age, with adult onset reported by 26% of patients with AD [6]. Although AD often resolves during childhood, it persists through adulthood in 20%-50% of patients [7,8].

AD is associated with substantial morbidity and quality of life (QoL) impairment. There are several comorbid health problems that occur in patients with AD, aside from the cutaneous signs and symptoms. Chronic pruritus and inflammation can lead to sleep disturbances and mental health symptoms, which are not mutually exclusive. AD may also predispose to a higher risk of other atopic disorders, including asthma and allergic rhinitis [9]. Persons with AD appear to be at higher risk for multiple neuropsychiatric disorders, including depression, anxiety, attention-deficit hyperactivity disorder, speech disorders in childhood, headaches, and seizures [10]. There is also a multifactorial association of AD with osteoporosis, bone and joint injuries, infections, and fractures [11-14].

Clinical presentation and severity of AD vary widely, and diagnosis is not always straightforward, especially in adults [15]. Treatment of AD follows a multifaceted, stepwise approach that is tailored according to disease severity [1]. For all patients, basic management and flare prevention consist of good skin care practices (daily showers or baths) followed immediately by the application of emollients and moisturizers, with avoidance of triggers such as irritants; aero or food allergens; and extremes of heat, cold, or humidity [9]. In mild AD, treatment involves, as needed, use of low- to mid-potency topical corticosteroids or topical calcineurin inhibitors during flares. Patients with frequent flares may benefit from proactive application of topical anti-inflammatory therapies twice a week to the most troublesome areas. Patients with severe disease often present significant treatment challenges. Systemic therapies are usually required for severe AD but have varying degrees of success and can be associated with side effect profiles that require counseling and close monitoring. Phototherapy has been shown to have success in treating moderate-to-severe AD, but several factors can limit its utility and efficacy including cost and access. New therapies are targeting specific pathways relevant for AD and many others are in development. An array of topical, oral, and injectable therapies targeting specific disease pathways in AD are in development for pediatric and adult populations [16]. Dupilumab (Dupixent) is the current first-line systemic agent for adults and children with moderate-to-severe, treatment-resistant AD. It is the only biologic treatment approved from 6 years of age. There are several emerging therapies currently in Phase III clinical trials such as JAK1 and JAK2 inhibitors or other biologic treatments (monoclonal antibodies or NK-1R antagonists) [16,17].

Social media is one of the most rapid and impactful ways of obtaining and delivering information in the modern era. In general, social media refers to forms of electronic communication (such as websites for social networking and microblogging) through which users create online communities to share information, opinions, personal messages, photos, videos, and other contents within internet applications [18]. Social media provides a readily accessible means to promote user-generated content, broaden interpersonal connections, and encourage social collaboration.

Social media has gained unprecedented worldwide popularity over the last 2 decades. It is estimated that there are currently over 2.3 billion active social media users internationally and this number is growing by approximately 1 million new users every day [19]. Unsurprisingly, therefore, the use of social media to find, exchange, and discuss health information is growing at an unprecedented rate. Anyone with access to the internet can post or read information on a (social media) site. This means that it is directly accessible to patients, their family and friends, and all health care providers. They provide access to active communities of health care professionals and fellow patients, with whom they can share information and their experiences, raise awareness of their concerns, learn about their conditions and health care opportunities, and find support [18].

Artificial intelligence (AI) tools are strongly related to data mining and AI is nowadays ranked among the top-10 technology [20]. Despite their limitations, AI tools and techniques that are still in their infancy already offer substantial benefits in providing in-depth knowledge on individuals’ health and predicting population health risks, and their use for medicine and public health is likely to increase substantially in the near future [21].

The purpose of this retrospective study was to better understand how patients suffering from AD perceive their QoL and their treatments, based on the assessment of web-based social media posts, which we considered as a real-life source of health information. In this study, we aimed to (1) identify the main themes for discussion; (2) identify the difficulties encountered by patients with respect to AD and the impact of the disease on QoL (physical, psychological, social, or financial); and (3) study the perception of patients regarding their treatment.

## Methods

### Study Design and Population

**Figure 1:**
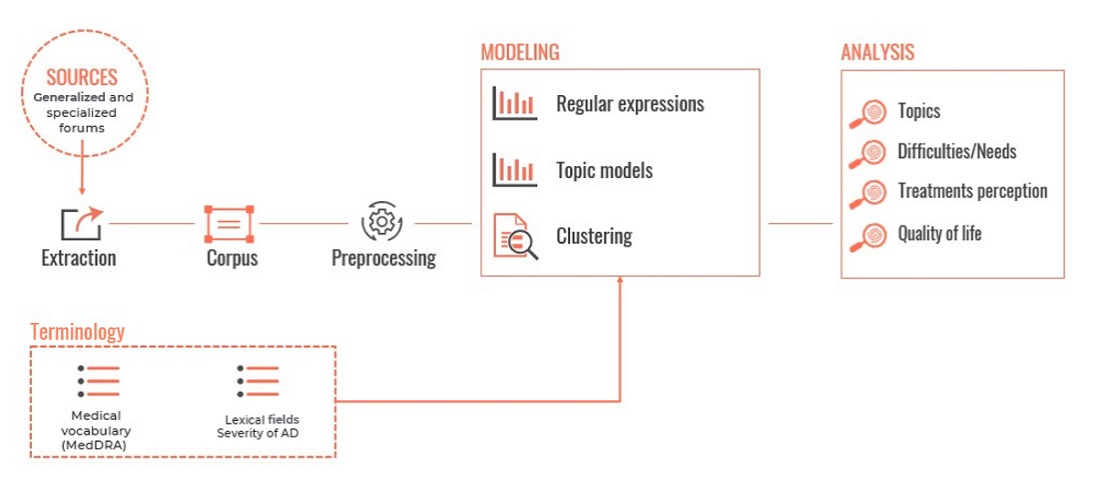
Study framework. AD: atopic dermatitis.

This was a noninterventional retrospective study using a text mining approach to retrieve information from social media posts (data available in the public domain) written by French language internet users between July 1, 2010, and October 23, 2020 (Figure 1). The study was conducted in 2 phases: data collection using the published Detec’t web crawler [22,23] developed by Kap Code to collect AD-related posts, and a quantitative/qualitative analysis to identify trends and characterize key themes discussed by French-speaking users.

### Data Extraction

A web crawler is an engine that browses through hyperlinks and stores them for future download of associated web pages (identified by the visited hyperlinks) [24]. Scraping of messages was performed according to the HTML structure of each forum. All discussions containing at least one of the keywords or one of their synonyms were automatically retrieved with all the associated metadata, deidentified, and cleaned (signature and quote withdrawal) before being stored in a study-specific database. A list of the keywords used for message retrieval is detailed in Multimedia Appendix 1 and a list of secondary filters on nonspecific extraction words is detailed in Multimedia Appendix 2. These terms were searched in extracted messages with extraction words not specific to AD. If one of these words was found in the post, then the message was kept in the corpus.

**Figure 2:**
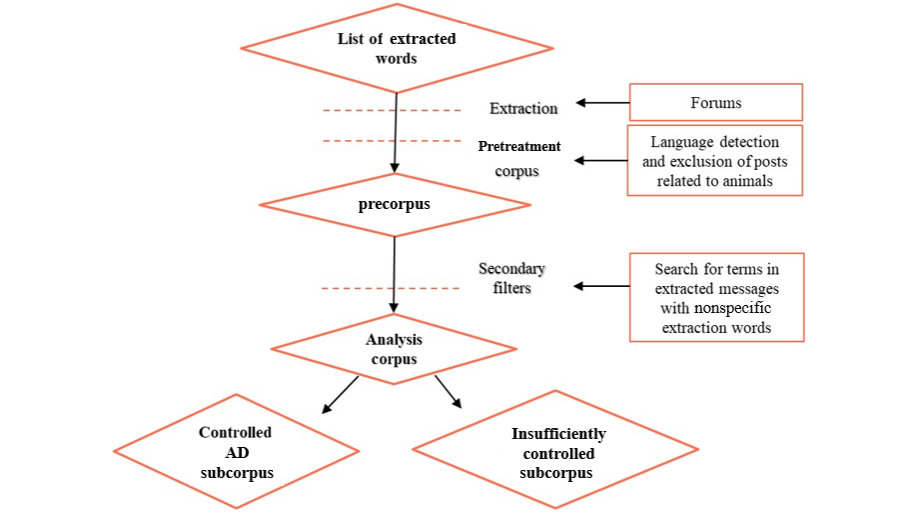
Flowchart presenting the steps for creating analysis corpus and the 2 subcorpuses. AD: atopic dermatitis.

The analysis corpus consisted of the corpus cleaned after the removal of messages containing predetermined keywords written in a language other than French, posts containing animal-related vocabulary, and messages containing at least one of the study-specific inclusion words listed in Multimedia Appendix 2. The analysis corpus was then divided into 2 subcorpuses: 1 subcorpus for *controlled AD* and 1 subcorpus for *insufficiently controlled AD* (Figure 2). The lexical fields of both subcorpuses were realized based on the Atopic Dermatitis Control Tool (ADCT) questionnaire and completed with real expressions of internet users [22].

### Data Analysis

#### Age and Gender

Web users’ gender was determined through the identification of regular expressions for each gender. First names and gender-associated suffix and prefix were first searched in the username. Then, the content of all available messages was screened for gender-specific lexical fields and gender agreements of adjectives and verbs. In the end, a score was computed for each gender and a prediction for the user was obtained by comparing them.

Ages of web users were identified by a twofold method. First, regular expressions of age were identified in the entire user post. If no expressions were found, then a machine learning model predicted the age based on several features. Among them, the model considered syntactic aspects of posts as well as expressed feeling and the source on which the users express themselves.

#### Topic Model

A topic model was applied to identify the themes addressed in the messages. Topic models consist of a text mining approach aiming to automatically identify the abstract themes addressed in a collection of documents. Such models are based on the hypothesis that each document in the corpus corresponds to a distribution of several topics. A Biterm Topic Model (BTM) was used to identify the topics without prior knowledge. A topic is defined as a subject of discussion, which amounts to tokens that frequently appear together in a corpus. The BTM considers the whole corpus as a mixture of topics, where each co-occurring pair of tokens (the biterm) is drawn from a specific topic independently and modeled topics are probability distributions over the biterms.

As topics are probability distributions over tokens of the study corpus, they can be characterized by the highest per-topic probability tokens. Weighting these probabilities through term-frequency inverse document frequency (TF-IDF) weighting allows to allocate a higher importance to topic-specific tokens. In this case, the per-topic probability of a token was weighted by the inverse of the probabilities of this token in other topics. For each topic, tokens were ranked from the highest to the lowest weighted probabilities TF-IDF value in this topic. The first 15 tokens were designated as the set of characteristic tokens and used to manually name the topic. A topic model was applied to each of the subcorpuses.

#### Health-Related Quality of Life

A health-related quality of life (HRQoL) algorithm was applied to identify and qualify expressed impact of the disease or treatments on the QoL of a patient. Types of impact were defined according to HRQoL survey categories. The algorithm was twofold: it indicates if an impact is expressed and, thanks to 5 specific models, it indicates the nature of the impact: physical, psychic, activity-related, relational, or financial. Features involved in the model describe expressed emotions, grammar, conjugation, and lexical fields of HRQoL-related features. The HRQoL algorithm was applied to each of the subcorpuses.

#### Drug Intake

To identify drug intake by the author of the message, the first step consisted of identifying the treatments cited in the 2 subcorpuses of messages. These were detected from the Detec’t database, which contains around 2500 molecules and drugs. After that, a prediction was made the simultaneous presence of the drug name and its associated adverse reaction in the same message (ie, a product–message couple). The model bases its prediction on regular forms expressing the intake of a treatment and their distance to the drug mention. Expressions were scored according to their probability of implying a treatment intake.

#### Feeling Analysis

In both subcorpuses, the content of the drug intake messages was analyzed (sentiment analysis) using Microsoft Azure Cognitive Services. Sentiment analysis is part of text analytics and can detect the level of positive or negative sentiment of input text using a confidence score across a variety of languages. This machine learning algorithm assigns a sentiment score to each sentence of a message, and then calculates the overall score to assign the message to a category (positive, negative, mixed, or neutral).

The sentiment analysis algorithm was applied to each of the drug intake messages’ subcorpuses. For drugs with few identified intakes, sentiment analysis was applied to treatment citation instead of treatment intake: this was the case for Dupixent for example.

The qualitative content of the positive and negative feelings in the messages was characterized by a manual identification with a review of posts detected with a treatment taken by the web users. The percentage of messages was calculated according to the total number of messages identified with terms related to treatments taken.

## Results

### Description of the Population and Posts

#### Overview

**Figure 3:**
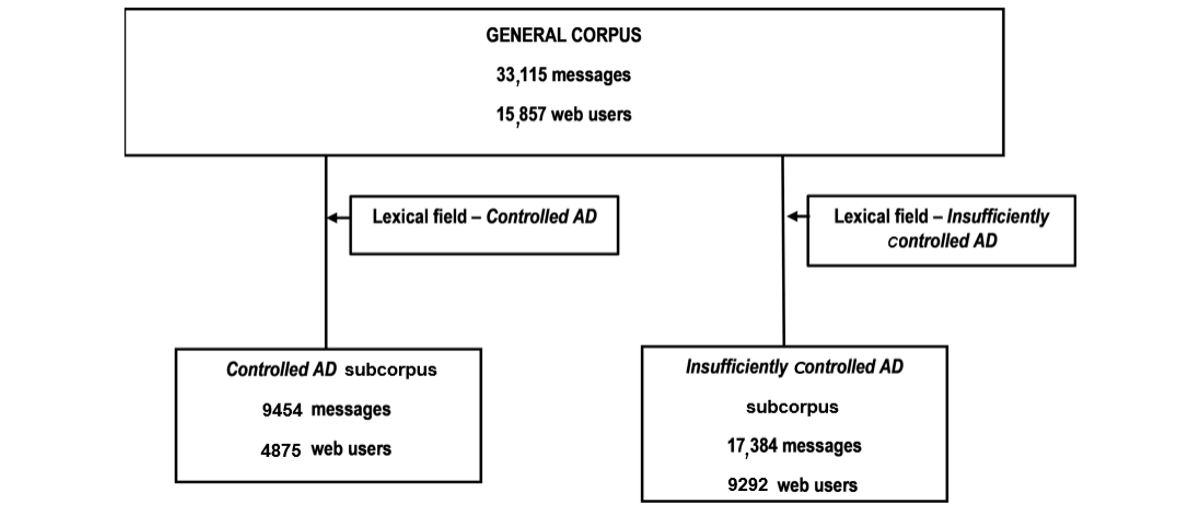
Methodology. AD: atopic dermatitis.

After cleaning and formatting, the analysis corpus contained a total of 33,115 messages corresponding to 15,857 different web users, with a median of 2.1 posts per patient (SD 9.55) (Figure 3). The *controlled AD* subcorpus contained 9454 posts corresponding to 4875 web users and the *insufficiently controlled* *AD* subcorpus contained about twice as many messages and web users with 17,384 posts from 9292 web users who published fewer messages than the other subcorpus (median of 1.87 posts per patient versus 1.94).

For both subgroups, extracted data mostly came from Baby Center [25] with 889/9454 (9.40%) posts for the *controlled AD* subcorpus and 2475/17,384 (14.24%) posts for the *insufficiently controlled AD* subcorpus (Table 1). The most frequently identified keyword was “Eczema” (7717/9454 posts for the *controlled AD* subcorpus and 13,039/17,384 posts for the *insufficiently controlled AD* subcorpus) followed by “Dermatite” (1146/9454 posts for the *controlled AD* subcorpus and 2386/17,384 posts for the *insufficiently controlled AD* subcorpus).

**Table 1:** Top 10 sites.

Controlled AD^a^			Insufficiently controlled AD		
Forums	Posts, n	Forums		Posts, n	
Baby Center	889	Baby Center		2475	
Aufeminin	307	Twitter		2431	
L’Appart’ des Spasmos	230	L’Appart’ des Spasmos		1288	
Amazon	220	Aufeminin		893	
Mamandco	206	Mamandco		385	
Twitter	128	Boursorama		282	
e-Sante	109	Amazon		228	
Forum Melodie	88	Mjeuxvideo		197	
Club Beaute Addict	61	Forum manucure		126	
Journal des Femmes	38	BourseReflex		121	

^a^AD: atopic dermatitis.

**Figure 4:**
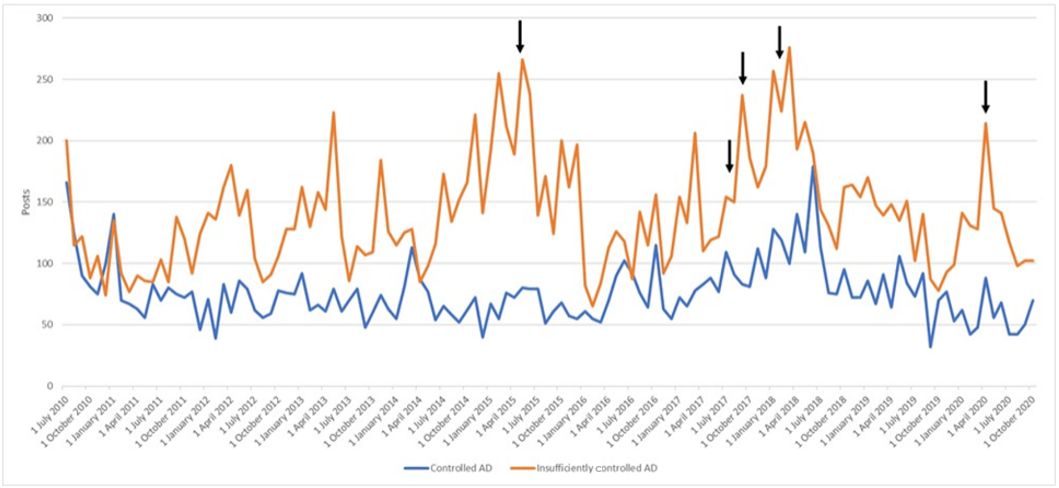
Fluctuations in the evolution of the volume of messages between July 2010 and October 2020. AD: atopic dermatitis.

We observed fluctuations in the evolution of the volume of messages between July 2010 and October 2020 (Figure 4) due to either seasonality or publications of scientific results related to AD. An apparent recurrent increase in the number of messages was observed from November to July that could be related to the increase in dust mites’ allergies in winter and the discomfort associated with sweating in summer [26,27]. From February to December 2015 there was a significant increase in the number of posts, which seems to be related to the publication of different scientific articles especially concerning the results of tofacitinib citrate, an oral Janus kinase inhibitor. Another increase in messages was also seen in 2018 that can be associated with the availability of Dupixent (dupilumab) in hospitals.

**Figure 5:**
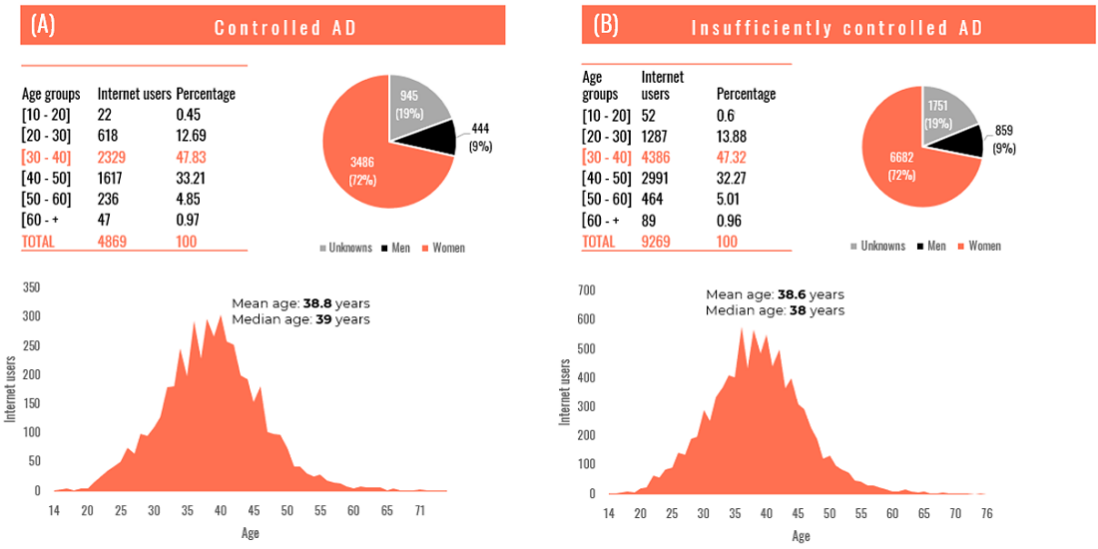
Age and gender distribution among (A) controlled AD subcorpus and (B) insufficiently controlled subcorpus. AD: atopic dermatitis.

In both subcorpuses, most users were women (3486/4869, 71.60%) with an average age of about 38 years and 19.41% (945/4869) appeared with undetermined gender (Figure 5). This was consistent with the results of many studies that point out that women express more personal issues in social networks [28,29]. In the present case, the topic of the disease was much discussed by mothers of children with AD who tended to focus on the appearance of their child’s skin.

**Figure 6:**
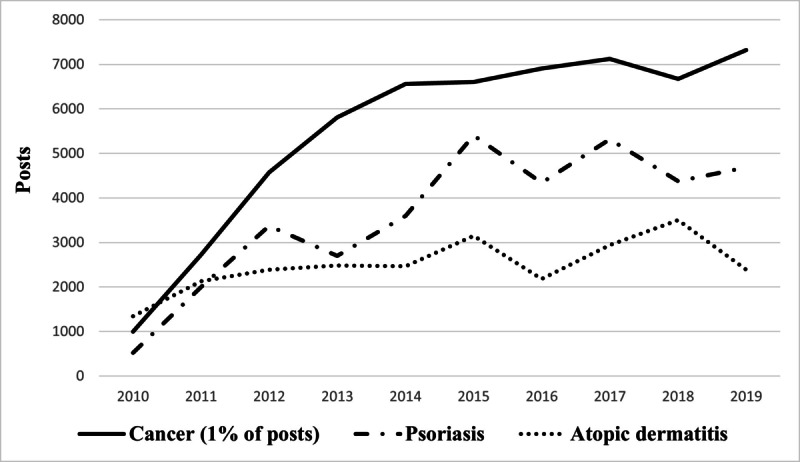
Trend over time of the number of posts concerning atopic dermatitis compared with other diseases such as cancer and psoriasis.

As shown in Figure 6, patients with AD are increasingly active online and use social media to participate, share their concerns, and express themselves freely regarding their disease. This trend was comparable to that of patients with psoriasis, even if the number of messages was lower for AD that was not sufficiently prioritized. By contrast, the comparison with the cancer disease area (1% of the messages) clearly highlighted the lack of specific forums or associations for AD compared with the cancer field.

#### Discussion Themes and Topics

**Figure 7:**
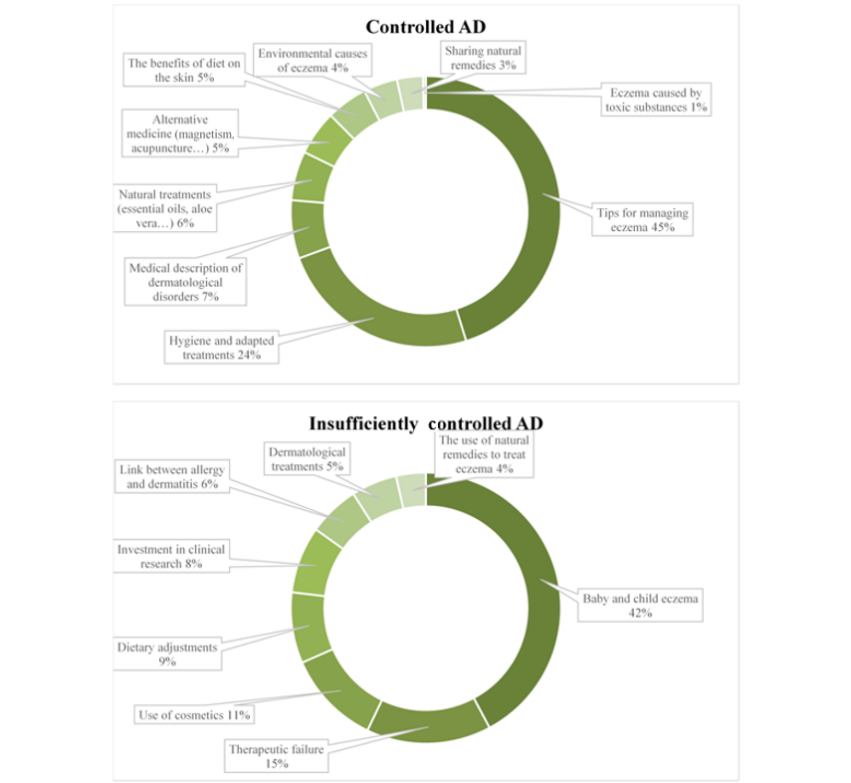
Distribution of posts by overall topic category. AD: atopic dermatitis.

Basically, the topics around AD were roughly the same between the 2 subcorpuses and the observed differences were due to the stage of the disease. Patients in the *controlled AD* subcorpus had more perspective and experience, whereas patients in the *insufficiently controlled AD* subcorpus lacked experience and were looking for information. As shown in Figure 7, the largest category of topics was related to “the exchange of tips to relieve eczema” (2864/6339, 45.18%), followed closely by “hygiene and care adapted with the disease” (1535/6339, 24.22%) for the *controlled AD* subcorpus. For the *insufficiently controlled AD* subcorpus, 42.19% (6351/15,055) of the discussions were about “eczema in babies and children” with most messages related to discussions between parents who are concerned about their child’s eczema and who ask questions to other web users. The second most discussed topic, with 14.93% (2247/15,055) of messages in the *insufficiently controlled* *AD* subcorpus, was the therapeutic failure experienced by web users. Their concerns reflected the poor knowledge of their disease and treatments. They expressed their frustration and lack of satisfaction about the treatments and solutions they have tested to reduce their symptoms. In the *controlled AD* subcorpus, by contrast, web users exchange information about natural remedies and alternative medicines to treat their AD. They expressed their satisfaction with the use of essential oils, food supplements, and other nonmedicinal methods and the effectiveness of these solutions. They used social networks to provide advice based on their own experience.

#### Quality of Life

The QoL is an important tool for evaluating the effect of a disease as well as effects of treatment interventions. In this study, the impact of AD on the QoL of individuals was assessed by monitoring physical well-being, social well-being, material well-being, emotional well-being, and development/activity.

**Figure 8:**
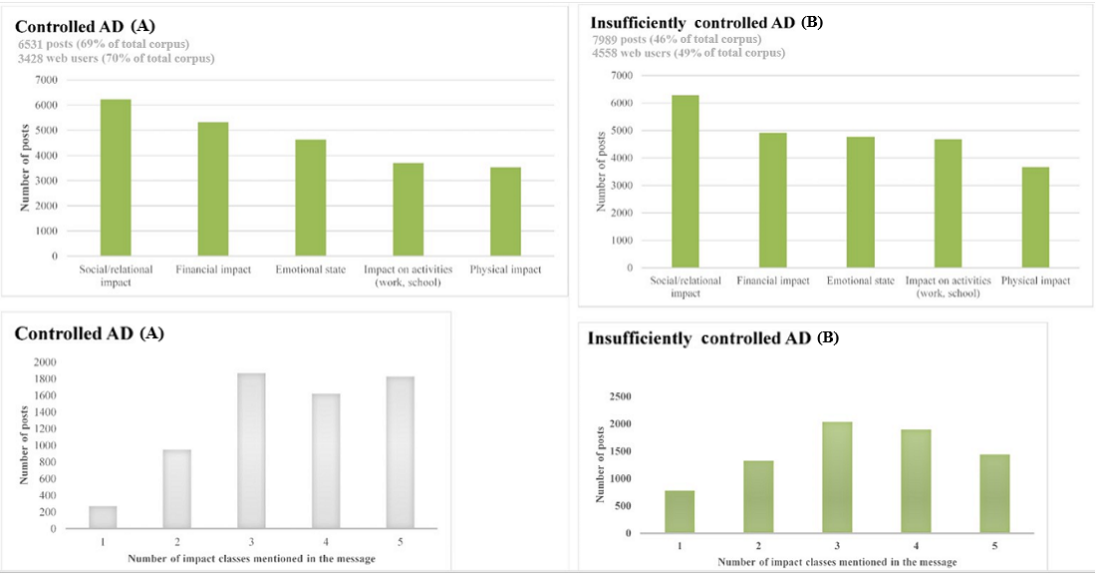
(A) Impact of the disease on controlled AD subcorpus. (B) Impact of the disease on insufficiently controlled AD subcorpus. AD: atopic dermatitis.

The impact of the disease on QoL was discussed by 70.32% (3428/4875) of patients in the *controlled AD* subcorpus (Figure 8A) and 49.05% (4558/9292) in the *insufficiently controlled AD* subcorpus (Figure 8B). This lower proportion can be explained by the heavy burden that AD places on patients with *insufficiently controlled AD*, particularly those who search for a solution to improve their health rather than their QoL. Furthermore, this trend can be related to the lack of awareness about their disease compared with patients from the *controlled AD* subcorpus, who have more knowledge about AD and therefore speak about their lived experience.

Patients in both subcorpuses seem to report the same impacts in their messages, but with a time lag that corresponds to the evolution of the knowledge of their disease.

Social functioning is affected by AD and is the first self-reported burden in both subcorpuses. Social isolation can be seen in young children with AD because adults and other children avoid interacting with them. The main effect of AD on the lives of adult patients stems from embarrassment and not wanting to be seen in public. Furthermore, some web users reported that they have been teased or bullied because of their AD. This trend was also reported by the International Study on Life with Atopic Eczema (ISOLATE) that found major impacts of AD on self-esteem [30].

The impacts that follows are the emotional impact (behavioral problems, irritability, crying, problems with treatments), the impact on activities (interference with activities such as bathing, swimming, and playing; clothing restrictions; decreased productivity at work; absenteeism), the impact on physical health (including itching, scratching, pain), and the economic impact (over-the-counter pharmacy costs among others). Independent of the corpus, there were on average 3 different impacts per message, which show the importance of this disease in everyday life.

### Treatments

AD treatment is targeted at both the disease and its symptoms. Among the 9454 messages retrieved from users in the *controlled AD* subcorpus, 1229 concerned discussions on the different treatments taken by the patients (ie, 13%). A focus was made on the top 10 and it appeared that web users in the *controlled AD* subcorpus were mainly focused on cortisone/corticosteroids and anti-inflammatory therapy. In addition, patients were prescribed antihistamines to decrease the itch–scratch cycle and antibiotics if the skin becomes superinfected (Table 2, data pertaining to “Controlled AD subcorpus”). Among the 17,384 messages retrieved from users in the *insufficiently controlled AD* subcorpus, 1677 concerned discussions on the different treatments taken by the patients (ie, 9.65%). Topically applied corticosteroids and emollients, moisturizers, and bath additives were the mainstay of therapy used by web users in the *insufficiently controlled AD* subcorpus followed by anti-inflammatory therapy to control pruritus and antihistamines as a therapeutic adjunct to alleviate it. In this subcorpus, treatments for acute phases such as topical corticosteroids, for example (Diprosone), were also discussed by patients from the *insufficiently controlled AD* subcorpus (Table 2, data pertaining to “Insufficiently controlled AD subcorpus”).

Then, the perception of treatments was found in 63.30% of the messages related to treatments taken by users in the *controlled AD* subcorpus (778/1229 messages) and in 72.09% of the messages dealing with treatments taken by users in the *insufficiently controlled AD* subcorpus (1209/1677 messages). In both subcorpuses, the most expressed treatment perception was cortisone.

**Figure 9:**
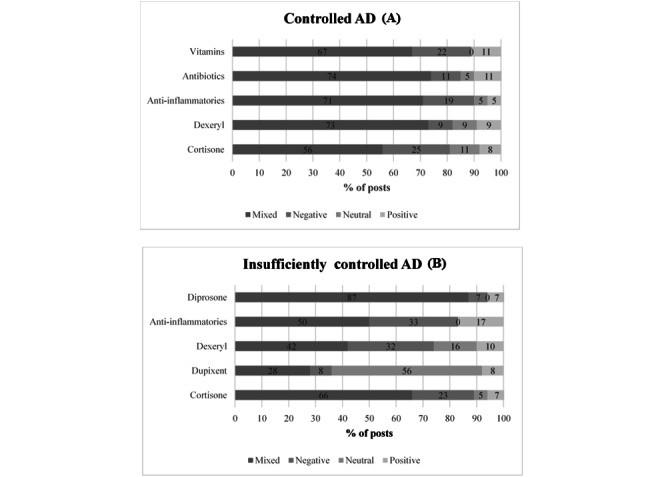
Treatments’ perception. (A) Controlled AD subcorpus. (B) Insufficiently Controlled AD subcorpus. AD: atopic dermatitis.

Mixed perception was found in 57% (47/83) of the *controlled AD* and 66% (114/173) of the *insufficiently controlled AD* with some concerns on the risks/side effects of cortisone emphasized by its efficacy. The perception was mostly negative (21/83, 25%) for *controlled AD* and 23% (39/173) for *insufficiently controlled AD* subcorpuses and positive in only 8% (7/83) of web users in the *controlled AD* and in 7% (12/173) of web users in the *insufficiently controlled AD* (Figure 9) subcorpuses. It is to note that in the lower panel, Dupixent is not a medication taken by patients with *insufficiently controlled AD* but only cited in their messages on which an analysis of perception was applied.

**Figure 10:**
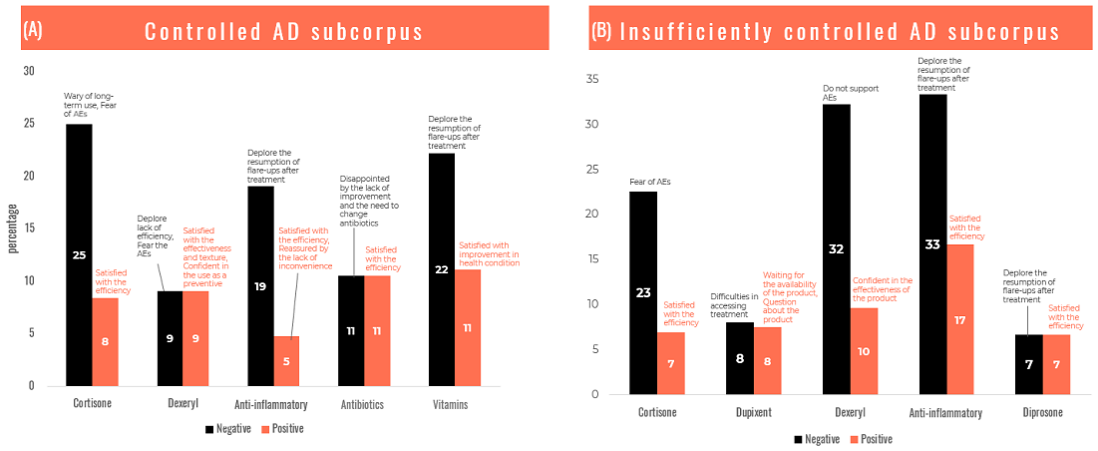
Treatments’ perception. (A) Controlled AD subcorpus. (B) Insufficiently controlled AD subcorpus. AD: atopic dermatitis; AE: adverse event.

Concerning cortisone, the *controlled AD* subcorpus and the *insufficiently controlled AD* subcorpus expressed safety concerns associated with their long-term use with negative feelings and beliefs (25% [21/83] and 23% [39/173] of the messages, respectively), whereas 8% (7/83) of users in the *controlled AD* subcorpus and 7% (12/173) of those in the *insufficiently controlled AD* subcorpus felt a positive perception following the effectiveness they had experienced. Positive and negative perceptions were quite counter-balanced between side effects and satisfaction for the texture and the use as a preventive treatment in patients with *controlled AD* using Dexeryl (9% [2/22] of positive and negative messages), whereas patients from the *insufficiently controlled AD* subcorpus reported more concerns about safety (10/31, 32%). The ineffectiveness of anti-inflammatories to calm the flare-ups was a concern for 19% (4/21) of users in the *controlled AD* subcorpus and 33% (6/18) of users in the *insufficiently controlled AD* subcorpus, whereas only 5% (1/21) and 17% (3/18), respectively, had a positive efficacy feeling. For Dupixent, there was an absence of messages from internet users who have taken or are aware of the treatment, and this may be due to its restricted prescription by dermatologists or directly from hospital and because it is not prescribed as first intention AD therapy. The only messages found were from patients seeking information mainly on the conditions of access (13/165, 7.9%) and on the effectiveness of the drug (13/165, 7.9%; Figure 10).

**Table 2:** Top 10 treatments used.

Controlled AD^a^ subcorpus		Insufficiently controlled AD subcorpus	
Treatments taken (top 10)	Web users, n	Treatments taken (top 10)	Web users, n
Cortisone/corticoids	131	Cortisone/corticoids	222
Anti-inflammatories	32	Liniment	40
Dexeryl	28	Dexeryl	39
Calendula	28	Anti-inflammatories	26
Liniment	26	Calendula	26
Antibiotics	26	Antibiotics	17
Vitamins	12	Diprosone	16
Atarax	8	Bepanthen	11
Bepanthen	6	Aerius	10
Aerius	5	Vitamins	10

^a^AD: atopic dermatitis.

## Discussion

### Principal Findings

This retrospective study aimed at assessing how patients with AD or their parents perceive their QoL and their treatments following the analysis of web forums in France over the last 10 years. AD is increasingly discussed in French web forums as shown in this study (Figure 6), with most users being women between the ages of 30 and 40 (Figure 5).

We were able to segment web users into 2 subcorpuses corresponding to patients with *controlled AD* and patients with *insufficiently controlled AD*. These subcorpuses are characterized by similarities in the expressions. Web users from the *controlled AD* subcorpus and those from the *insufficiently controlled AD* subcorpus have almost the same preoccupations but we observed a time lag due in part to the stage and the knowledge of their disease. The main topics were the lack of information and solutions leading to some sort of therapeutic wandering, the high impact of AD on the QoL and the well-being of patients, and the dissatisfaction with the available treatments.

### Limitations of the Study

Given the inherent observational nature of social media data, their analyses are subject to many limitations.

Selection bias was the first limitation because analyses were restricted to French data sources, and social media users and nonusers may differ. Thus, results are not generalizable at a worldwide scale. Furthermore, using social media to analyze patients’ reactions excludes patients who do not have access to the internet or who are not familiar with the use of online discussions.

Extraction bias was the second limitation. Keyword selection in social media studies can induce varying levels of extraction bias.

Another limitation of using social media is that complete information about individual cases may be harder to obtain, unlike traditional epidemiological studies. There is also the problem of discovering demographic information—only limited or no information regarding individual user demographics may be available. Demographic information such as age and race need to be determined via automated techniques.

Health misinformation is significant on social media; nevertheless, recent reviews showed that misinformation or fake information related to health is most prevalent in studies dealing with the safety of tobacco, vaccines, and drugs such as opioids and marijuana. Finally, the lowest levels of misinformation were observed in studies evaluating medical treatments [31,32]. Nevertheless, results from Pulido et al [33] indicated that messages focused on fake health information are mostly aggressive and that messages with evidence of social impact overcome fake information.

Finally, social media represent an ideal place where patients can freely and spontaneously discuss their experiences with their therapy, thus providing valuable information on their QoL. However, this observation should be interpreted cautiously, because social media data may include a higher frequency of erroneous information, and patients posting on social media forums may not be representative of the wider patient population.

### Implications and Future Research

Messages published on social networks should be integrated into the assessment of patient’s QoL, as they can help to characterize the patient’s experience in a more individualized and spontaneous way. Furthermore, it seems important to explore the specific areas of QoL of patients with AD and potentially enrich the existing standard questionnaires with new elements more relevant for these patients in their daily confrontation with disease and treatment.

To the extent that the most dominant topics can be interpreted as unmet informational needs, our study highlights the refinement of practical implications such as the improvement of available tools, and further analysis on the perception of treatments and the evolution of the QoL under treatment. We recommend to carry out a study segmented by year to see the evolution of the different subjects (QoL, topics, treatments) over the years and to observe the global evolutions.

### Conclusion

Social media listening offers the opportunity to consider behavior and interactions that are difficult to assess through traditional research methods. Millions of microblogs act as online communities, dealing with topics from the impact of AD on QoL through the lack of information on possible treatments and their effectiveness. Of particular interest is the exponential growth in recent years of patient support groups, and the high potential of users disseminating materials and opinions relating to AD through their posts. These forums are increasingly popular and have become an additional source of evidence, therapy, or support for the patients. Our study illustrates the current situation and the evolution over time of AD on social media platforms. Discussions on AD are becoming a frequent purpose on social media but they are still in contrast to the high frequency reported for the use of social media in research with patients with psoriasis.

All these data highlight the importance of clearly defining the role and limitations of these platforms for orienting future information campaigns and developing new models such as the ADCT centered on patients with AD [34]. This validated 6-item ADCT facilitates patient–physician communication on disease control. Such a tool might better inform health care professionals and patients with individualized measures covering QoL more in depth than existing standard questionnaires.

## Appendix

Multimedia Appendix 1 List of keywords used for the extraction of messages.

Multimedia Appendix 2 Number of extracted messages and associated number of web users per data source.

## Abbreviations

AD: atopic dermatitis
ADCT: Atopic Dermatitis Control Tool
AI: artificial intelligence
BTM: Biterm Topic Model
HRQoL: health-related quality of life
ISOLATE: International Study on Life with Atopic Eczema
QoL: quality of life
TF-IDF: term-frequency inverse document frequency
