# Supplementary material for: Social Media Platforms Listening Study on Atopic Dermatitis: Quantitative and Qualitative Findings
Source: J Med Internet Res. 2022 Jan 28;24(1):e31140. doi: 10.2196/31140 (PMC8838596; doi:10.2196/31140)
Supplement: Multimedia Appendix 1 [file jmir_v24i1e31140_app1.docx]

Appendix 1 - List of keywords used for the extraction of messages.

| Affection inflammatoire prurigineuse |
| --- |
| Crème à la cortisone* |
| Crème corticoide* |
| Dermatite |
| Dermatite atopique |
| Dermatite atopique du nourisson |
| Dermatite atopique modérée |
| Dermatite atopique modérée à severe |
| Dermatite atopique sévère |
| Dermatose chronique prurigineuse |
| Dermocorticoide* |
| Dupilumab |
| Dupixent |
| Eczema* |
| Eczema atopique |
| Eczema constitutionnel |
| * Non-specific extraction words for which a secondary filter search has been performed |
|  |
|  |
|  |
|  |
|  |
|  |
|  |
|  |
|  |
|  |
|  |
|  |
|  |
|  |
|  |
|  |
|  |
|  |
|  |
|  |
|  |
|  |
|  |
|  |
|  |
|  |
|  |
|  |
|  |
|  |
|  |
|  |
|  |
|  |
|  |
|  |
|  |
|  |
|  |
|  |
|  |
