# Supplementary material for: Social Media Platforms Listening Study on Atopic Dermatitis: Quantitative and Qualitative Findings
Source: J Med Internet Res. 2022 Jan 28;24(1):e31140. doi: 10.2196/31140 (PMC8838596; doi:10.2196/31140)
Supplement: Multimedia Appendix 2 [file jmir_v24i1e31140_app2.docx]

Appendix 2 - Number of extracted messages and associated number of web users per data source.

| Gratte | Sommeil | Recouvert sur tout le corps | Da severe | Croute jaunatre |
| --- | --- | --- | --- | --- |
| Demangeaison | Plaque | Partout sur le corps | Plaque rouge | Lesion |
| Demange | Asthme | Dermatite | Plaques rouges | Pellicule |
| Suintement | Rhinite | Eczema atopique | Crevasse | Lichénification |
| Suinte | Hérédité | Dermatite atopique severe | Saignement | Grattant |
| Peau seche | Hérité | Dupilumab | Saigne | Rougeur |
| Secheresse | Héréditaire | Dupixent | Desquamation | Eruption cutane |
| Desseche | Temporalité depuis l’enfance | Dermatite atopique du nourisson | Dors plus | Dermatite atopique |
| Asseche | Depuis tout petit | Eczema constitutionnel | Dort mal | Croute jaune |
| Grattage | Depuis toute petite | Dermatite atopique moderee a severe | Dort pas |  |
| Peau rouge | Mal à dormir | Affection inflammatoire prurigineuse chronique | Dort plus |  |
| Visage rouge | Dors mal | Dermatose chronique prurigineuse | Tache rouge |  |
| Prurit | Dors pas | Dermatite atopique moderee | Taches rouges |  |
